# Supplementary material for: Three Dimensional Polarimetric Neutron Tomography of Magnetic Fields
Source: Sci Rep. 2018 Feb 2;8:2214. doi: 10.1038/s41598-018-20461-7 (PMC5797168; doi:10.1038/s41598-018-20461-7)
Supplement: Supplementary file 1 — Supplementary information [file 41598_2018_20461_MOESM1_ESM.pdf]

Supplementary Information – Three Dimensional Polarimetric Neutron Tomography of Magnetic Fields

Morten Sales[1,*], Markus Strobl[2,3], Takenao Shinohara[4], Anton Tremsin[5], Luise Theil Kuhn[6], William R.B. Lionheart[7], Naeem M. Desai[7], Anders Bjorholm Dahl[8], and Søren Schmidt[1,]**

[1]Department of Physics, Technical University of Denmark, DK-2800 Kgs. Lyngby, Denmark
[2]Laboratory for Neutron Scattering and Imaging, Paul Scherrer Institute, 5232 Villigen, Switzerland
[3]Niels Bohr Institute, University of Copenhagen, Copenhagen, DK-2100, Denmark
[4]J-PARC Center, Japan Atomic Energy Agency, Tokai 319-1195, Japan
[5]Space Sciences Laboratory, University of California at Berkeley, Berkeley, CA 94720, USA
[6]Department of Energy Conversion and Storage, Technical University of Denmark, DK-4000 Roskilde, Denmark
[7]School of Mathematics, The University of Manchester, Manchester, M13 9PL, United Kingdom
[8]Department of Applied Mathematics and Computer Science, Technical University of Denmark, DK-2800 Kgs. Lyngby, Denmark
[*]msales@fysik.dtu.dk
[**]ssch@fysik.dtu.dk

ABSTRACT

This document provides supplementary information to the article on Three Dimensional Polarimetric Neutron Tomography (3DPNT) of Magnetic Fields. Here, we present at detailed comparison of the reconstruction of the measured magnetic field from a current carrying solenoid with Biot-Savart calculations as well as reconstructions based on ray-tracing simulations.

A Supplementary details on reconstruction

We here compare the following four characterisations of the magnetic field generated by a current carrying solenoid:

1. Reconstruction from measurement.
   Our results from using three dimensional polarimetric neutron tomography to measure the magnetic field.

2. Biot-Savart calculation.
   (see eq. 11).

3. Reconstruction from ray-tracing simulated data.
   Using the Biot-Savart calculated field to generate simulated data, that is used for reconstruction.
   Same number of neutron paths (N=48), and projection angles (A=30), as in measurement.

4. Reconstruction from ray-tracing simulated data.
   Using the Biot-Savart calculated field to generate simulated data, that is used for reconstruction.
   Higher number of neutron paths (N=512), and projection angles (A=180).

The Ray-tracing (and reconstruction) was done for each slice, where the classical Rodrigues formula[1] was used to calculate the neutron spin precession. Neutrons in a parallel beam with a wavelength of 3 Å was used.

For each of the three curves (red, green, and blue) shown in Fig. A.1, the magnetic field is plotted for comparison between the different characterisations is plottet in Fig. A.2. It can be seen that a there's a high level of correspondence between all curves, however, with the results from the measurements generally showing slightly lower fields strengths. This is to be expected as the measured solenoid is certain to have a degree of imperfection compared to the perfect model used for calculation and simulation. From comparing simulations to calculations it can be seen that our reconstruction technique works well, though the level of detail in features of the quickly changing fields around the border of the solenoid is especially dependent on the resolution.

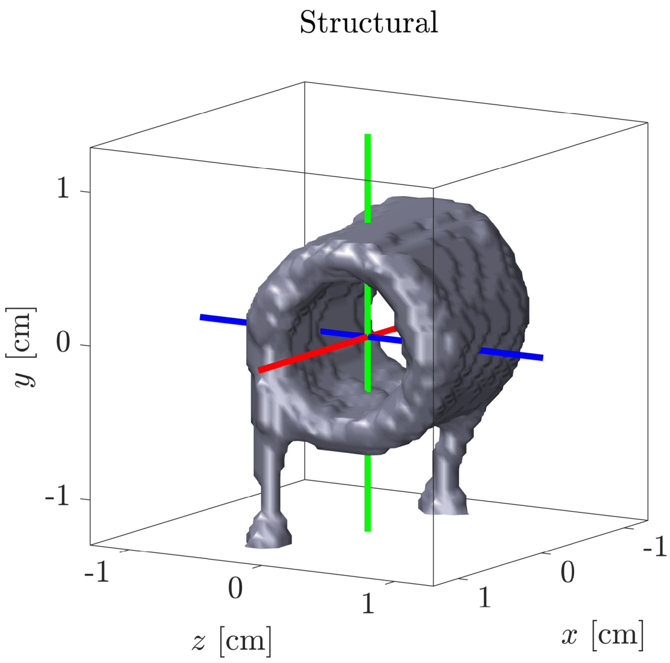

Figure A.1. Structural reconstruction with curves for which comparison is shown in Fig. A.2.

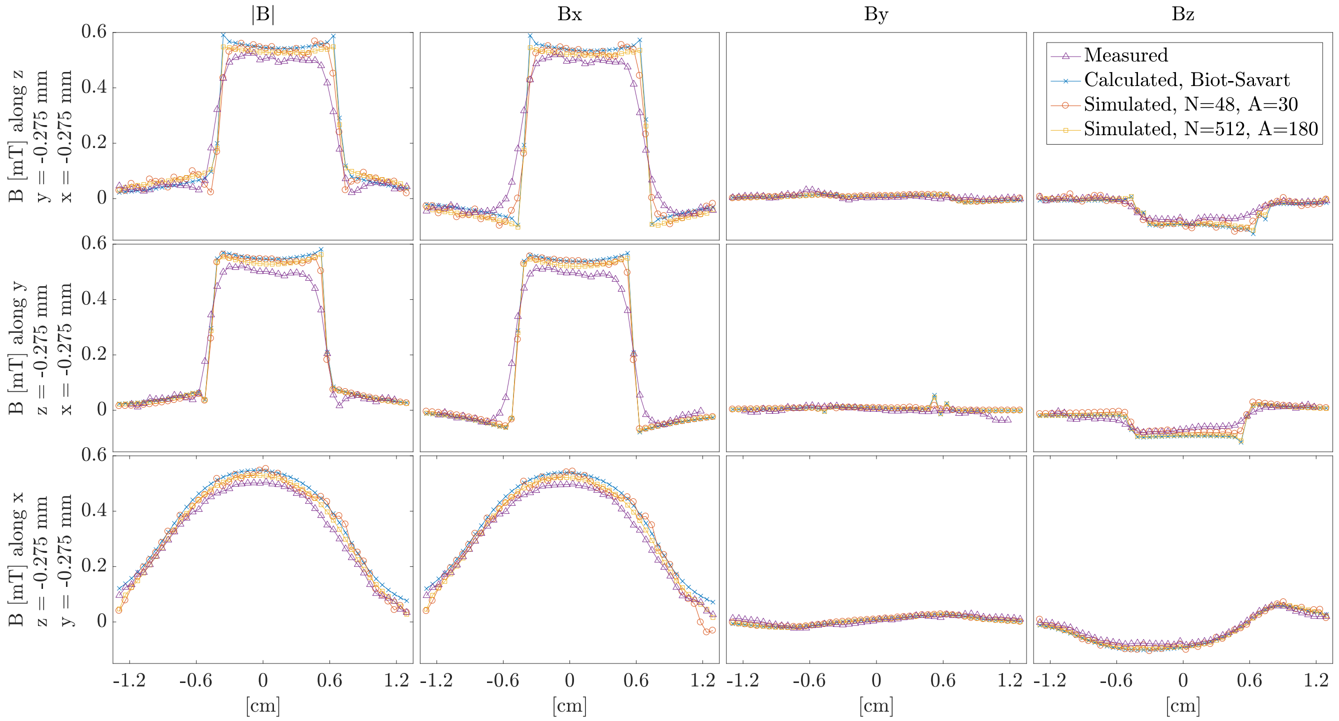

Figure A.2. Comparison between 3DPNT measured magnetic field generated by current carrying solenoid, to Biot-Savart calculation, as well as simulation based reconstructions with different resolution. The columns are the magnetic field strength and B_x, B_y, and B_z respectively. The rows correspond to the three different curves shown in Fig. A.1.

References

1. Rodrigues, B. O. Des lois geometriques qui regissent les deplacements d'un systeme solide dans l'espace, et de la variation des coordonnees provenant de ces deplacements consideres independamment des causes qui peuvent les produire. *J. des Math. Pures et Appliquees* **5**, 380–440 (1840)
